# Supplementary material for: NG2 glia protect against prion neurotoxicity by inhibiting microglia-to-neuron prostaglandin E2 signaling
Source: Nat Neurosci. 2024 May 27;27(8):1534–44. doi: 10.1038/s41593-024-01663-x (PMC11303249; doi:10.1038/s41593-024-01663-x)
Supplement: Supplementary file 1 — Reporting Summary [file 41593_2024_1663_MOESM1_ESM.pdf]

nature portfolio

Corresponding author(s): Yingjun Liu; Adriano Aguzzi

Last updated by author(s): Mar 12, 2024

Reporting Summary

Nature Portfolio wishes to improve the reproducibility of the work that we publish. This form provides structure for consistency and transparency in reporting. For further information on Nature Portfolio policies, see our Editorial Policies and the Editorial Policy Checklist.

Statistics

For all statistical analyses, confirm that the following items are present in the figure legend, table legend, main text, or Methods section.

n/a	Confirmed	
☐	☒	The exact sample size (*n*) for each experimental group/condition, given as a discrete number and unit of measurement
☐	☒	A statement on whether measurements were taken from distinct samples or whether the same sample was measured repeatedly
☐	☒	The statistical test(s) used AND whether they are one- or two-sided<br>*Only common tests should be described solely by name; describe more complex techniques in the Methods section.*
☐	☒	A description of all covariates tested
☐	☒	A description of any assumptions or corrections, such as tests of normality and adjustment for multiple comparisons
☐	☒	A full description of the statistical parameters including central tendency (e.g. means) or other basic estimates (e.g. regression coefficient) AND variation (e.g. standard deviation) or associated estimates of uncertainty (e.g. confidence intervals)
☐	☒	For null hypothesis testing, the test statistic (e.g. *F*, *t*, *r*) with confidence intervals, effect sizes, degrees of freedom and *P* value noted<br>*Give P values as exact values whenever suitable.*
☒	☐	For Bayesian analysis, information on the choice of priors and Markov chain Monte Carlo settings
☒	☐	For hierarchical and complex designs, identification of the appropriate level for tests and full reporting of outcomes
☒	☐	Estimates of effect sizes (e.g. Cohen's *d*, Pearson's *r*), indicating how they were calculated

Our web collection on statistics for biologists contains articles on many of the points above.

Software and code

Policy information about availability of computer code

Data collection	Membranes of western blots were digitalized with ImageQuant (LAS-4000, Fujifilm) using its associated software (Image Reader version 1.2). Fluorescent images were captured with Nikon Eclipse Ti2-E confocal fluorescent microscope and FLUOVIEW FV10i confocal microscope (Olympus Life Science) using their associated softwares (NIS-Elements AR 5.30.05 and FV10-ASW Ver.04.02, respectively).
Data analysis	ImageJ 1.53t was used to quantify band density of western blots and signal of fluorescent images. Graphpad Prism 9 and R version 4.3.1 were used for data visualization and statistical tests. R version 4.3.1 was used for analysis of single-cell RNA sequencing data with the following packages, Seurat 4.4.0, SeuratWrappers 0.3.1, harmony 1.1.0, CellChat 1.6.1.

For manuscripts utilizing custom algorithms or software that are central to the research but not yet described in published literature, software must be made available to editors and reviewers. We strongly encourage code deposition in a community repository (e.g. GitHub). See the Nature Portfolio guidelines for submitting code & software for further information.

Data

Policy information about availability of data

All manuscripts must include a data availability statement. This statement should provide the following information, where applicable:

- Accession codes, unique identifiers, or web links for publicly available datasets
- A description of any restrictions on data availability
- For clinical datasets or third party data, please ensure that the statement adheres to our policy

Single-cell RNA sequencing data were obtained from the single cell portal at the Broad institute through the link: https://singlecell.broadinstitute.org/single_cell/study/SCP1962. Other data associated with the findings of the current study are provided as Figures, Extended Data Figures, Supplementary Information or Source Data.

Research involving human participants, their data, or biological material

Policy information about studies with human participants or human data. See also policy information about sex, gender (identity/presentation), and sexual orientation and race, ethnicity and racism.

Reporting on sex and gender	N/A
Reporting on race, ethnicity, or other socially relevant groupings	N/A
Population characteristics	N/A
Recruitment	N/A
Ethics oversight	N/A

Note that full information on the approval of the study protocol must also be provided in the manuscript.

Field-specific reporting

Please select the one below that is the best fit for your research. If you are not sure, read the appropriate sections before making your selection.

☒ Life sciences ☐ Behavioural & social sciences ☐ Ecological, evolutionary & environmental sciences

For a reference copy of the document with all sections, see nature.com/documents/nr-reporting-summary-flat.pdf

Life sciences study design

All studies must disclose on these points even when the disclosure is negative.

Sample size	Sample sizes were based on literatures or our previous experiments (Liu et al., 2020, Glia; Liu et al., 2022, Communications Biology; Avar et al., 2022, the EMBO Journal; Zhu et al., 2019, Journal of Experimental Medicine), and were not determined by statistical methods in the current manuscript.
Data exclusions	No data were excluded from analysis.
Replication	In vivo experiments were performed with at least 3 biological replicates. All other experiments were performed with at least 3 biological and technical replicates. All attempts of replication were successful.
Randomization	In all experiments, mice, brain slices, cell cultures or other samples were randomly assigned to experimental conditions and time-points.
Blinding	The investigators were blinded for animal grouping, treatments and tissue collection for in vivo experiments, but were not blinded for some of the ex vivo experiments due to that blinding is practically infeasible or temporary shortages of manpower.

Reporting for specific materials, systems and methods

We require information from authors about some types of materials, experimental systems and methods used in many studies. Here, indicate whether each material, system or method listed is relevant to your study. If you are not sure if a list item applies to your research, read the appropriate section before selecting a response.

Materials & experimental systems

n/a	Involved in the study
☐	☒ Antibodies
☐	☒ Eukaryotic cell lines
☒	☐ Palaeontology and archaeology
☐	☒ Animals and other organisms
☒	☐ Clinical data
☒	☐ Dual use research of concern
☒	☐ Plants

Methods

n/a	Involved in the study
☒	☐ ChIP-seq
☒	☐ Flow cytometry
☒	☐ MRI-based neuroimaging

Antibodies

Antibodies used

mouse monoclonal antibody against actin (1:10,000, Merck Millipore, MAB1501R, clone C4); mouse monoclonal antibody against PrP (POM1, 1:5000, homemade); rabbit polyclonal antibody against NG2 (1:500, MERCK, AB5320); rabbit polyclonal antibody against PDGFRα (1:500, Santa Cruz, SC-338); rabbit monoclonal antibody against NeuN (1:1000, Abcam, ab177487, clone EPR12763); rabbit polyclonal antibody against NG2 (1:500, a gift from Prof. Stallcup); rabbit polyclonal antibody against Iba1 (1:500, Wako, 019-19741); Rat monoclonal antibody against Cd68 (1:200, BioRad, MCA1957, clone FA-11); rabbit polyclonal antibody against Map2 (1:200, Biolegend, 840601), mouse monoclonal antibody against Cox2 (1:200, Santa Cruz, sc-166475, clone D-12); mouse monoclonal antibody against Ptges (1:200, Santa Cruz, sc-365844, clone H-3); rabbit polyclonal antibody against EP1 (1:200, Bioss Antibodies, BS-6316R); rabbit monoclonal antibody against EP2 (1:200, Abcam, ab167171, clone EPR8030(B)); rabbit polyclonal antibody against EP3 (1:200, Cayman Chemical, 101760); mouse monoclonal antibody against EP4 (1:200, ProteinTech, 66921-1-Ig, clone 4A2A12); rat monoclonal antibody against CD11b antibody (30 ul in 10 ml, ThermoFisher Scientific, 14-0112-82, clone M1/70), mouse monoclonal antibody against Tau (1:200, ThermoFisher Scientific, MN1010, clone BT2), chicken polyclonal antibody against NeuN (1:1000, Merck, ABN91), goat polyclonal antibody against rat IgG (30 ul in 10 m, Jackson ImmunoResearch, 112-005-167); HRP-conjugated goat anti-rabbit IgG antibody (1:10,000, Jackson ImmunoResearch, 111-035-003); HRP-conjugated goat anti-mouse IgG antibody (1:10,000, Jackson ImmunoResearch, 115-035-003); Alexa488-conjugated goat anti-mouse IgG antibody (1:3000, ThermoFisher Scientific, A32723); Alexa594-conjugated goat anti-rabbit IgG antibody (1:3000, ThermoFisher Scientific, A32740); Alexa647-conjugated goat anti-chicken IgG antibody (1:3000, ThermoFisher Scientific, A32933).

Validation

All antibodies have been validated by the manufacturers and previous publications. For mouse monoclonal antibody against actin (Merck Millipore, MAB1501R, clone C4): https://www.merckmillipore.com/CH/de/product/Anti-Actin-Antibodyclone-C4,MM_NF-MAB1501R?ReferrerURL=https%3A%2F%2Fwww.google.com%2F; for mouse monoclonal antibody against PrP (clone POM1, homemade): Polymenidou et al., 2008, PloS One; for rabbit polyclonal antibody against NG2 (MERCK, AB5320): https://www.merckmillipore.com/CH/de/product/Anti-NG2-Chondroitin-Sulfate-Proteoglycan-Antibody,MM_NF-AB5320?ReferrerURL=https%3A%2F%2Fwww.google.com%2F; for rabbit polyclonal antibody against PDGFRα (Santa Cruz, SC-338): Liu et al., 2020, Glia; for rabbit monoclonal antibody against NeuN (Abcam, ab177487, clone EPR12763): https://www.abcam.com/products/primary-antibodies/neun-antibody-epr12763-neuronal-marker-ab177487.html; for rabbit polyclonal antibody against NG2 (a gift from Prof. Stallcup): Liu at al., 2020, Glia; for rabbit polyclonal antibody against Iba1 (Wako, 019-19741): https://labchem-wako.fujifilm.com/us/product/detail/W01W0101-1974.html; for at monoclonal antibody against Cd68 (BioRad, MCA1957): https://www.bio-rad-antibodies.com/monoclonal/mouse-cd68-antibody-fa-11-mca1957.html?f=purified; for rabbit polyclonal antibody against Map2 (Biolegend, 840601): https://www.biolegend.com/ja-jp/products/anti-map2-antibody-11086?GroupID=BLG15645; for mouse monoclonal antibody against Cox2 (Santa Cruz, sc-166475, clone D-12): https://www.scbt.com/p/cox-2-antibody-d-12; for mouse monoclonal antibody against Ptges (Santa Cruz, sc-365844, clone H-3): https://www.scbt.com/p/pge-synthase-antibody-h-3; for rabbit polyclonal antibody against EP1 (Bioss Antibodies, BS-6316R): https://www.biossusa.com/products/bs-6316r; for rabbit monoclonal antibody against EP2 (Abcam, ab167171, clone EPR8030(B)): https://www.abcam.com/products/primary-antibodies/prostaglandin-e-receptor-ep2ptger2-antibody-epr8030b-ab167171.html; for rabbit polyclonal antibody against EP3 (Cayman Chemical, 101760): https://www.caymanchem.com/product/101760/ep3-receptor-polyclonal-antibody; for mouse monoclonal antibody against EP4 (ProteinTech, 66921-1-Ig, clone 4A2A12): https://www.ptglab.com/products/PTGER4-Antibody-66921-1-Ig.htm; for rat anti-mouse CD11b antibody (ThermoFisher Scientific, 14-0112-82, clone M1/70): https://www.thermofisher.com/antibody/product/CD11b-Antibody-clone-M1-70-Monoclonal/14-0112-82; for mouse monoclonal antibody against Tau (ThermoFisher Scientific, MN1010, clone BT2): https://www.thermofisher.com/antibody/product/Tau-Antibody-clone-BT2-Monoclonal/MN1010; for chicken polyclonal antibody against NeuN (Merck, ABN91): https://www.merckmillipore.com/CH/de/product/Anti-NeuN-Antibody,MM_NF-ABN91?ReferrerURL=https%3A%2F%2Fwww.google.com%2F; for goat polyclonal antibody against rat IgG (Jackson ImmunoResearch, 112-005-167): https://www.jacksonimmuno.com/catalog/products/112-005-167; for HRP-conjugated goat anti-rabbit IgG antibody (Jackson ImmunoResearch, 111-035-003): https://www.jacksonimmuno.com/catalog/products/111-035-003; for HRP-conjugated goat anti-mouse IgG antibody (Jackson ImmunoResearch, 115-035-003): https://www.jacksonimmuno.com/catalog/products/115-035-003; for Alexa488-conjugated goat anti-mouse IgG antibody (ThermoFisher Scientific, A32723): https://www.thermofisher.com/antibody/product/Goat-anti-Mouse-IgG-H-L-Highly-Cross-Adsorbed-Secondary-Antibody-Polyclonal/A32723; for Alexa594-conjugated goat anti-rabbit IgG antibody (ThermoFisher Scientific, A32740): https://www.thermofisher.com/antibody/product/Goat-anti-Rabbit-IgG-H-L-Highly-Cross-Adsorbed-Secondary-Antibody-Polyclonal/A32740; for Alexa647-conjugated goat anti-chicken IgG antibody (1:3000, ThermoFisher Scientific, A32933): https://www.thermofisher.com/antibody/product/Goat-anti-Chicken-IgY-H-L-Cross-Adsorbed-Secondary-Antibody-Polyclonal/A32933.

Eukaryotic cell lines

Policy information about cell lines and Sex and Gender in Research

Cell line source(s)

HovS cells (Avar, Heinzer et al. 2020): a subclone of the human SH-SY5Y cell line, where the human PRNP gene was replaced with the ovine PRNP VRQ allele. HEK293T cells are from ATCC (CRL-3216).

Authentication	Cell lines were not authenticated.
Mycoplasma contamination	No Mycoplasma was detected.
Commonly misidentified lines (See ICLAC register)	No such cell lines were used.

Animals and other research organisms

Policy information about studies involving animals; ARRIVE guidelines recommended for reporting animal research, and Sex and Gender in Research

Laboratory animals	C57BL6/J mice were obtained from Charles River, Germany. Tga20 mice (Fischer, Rulicke et al. 1996) were obtained from Laboratory Animal Services Center at University of Zurich, Switzerland. Pdgfrα-CreER mice (Stock No: 018280), iDTR mice (Stock No: 007900) and Cox2Luc mice (Stock No: 030853) were obtained from the Jackson Laboratory, USA. Double transgenic Pdgfrα-CreER/iDTR mice were generated by crossing Pdgfrα-CreER mice with iDTR mice. To generate Pdgfrα/iDTR mice on a Cox2Luc background, Pdgfrα-CreER mice and iDTR mice were first crossed with the Cox2Luc mice, then the resulting Pdgfrα-CreER mice and iDTR mice on the Cox2Luc background were crossed with each other. Littermates of the experimental groups were used as controls. Postnatal day 12 pups were used for preparing brain slice cultures. Postnatal day 5 pups were used for primary microglia and neuronal cultures. For other experiments, the mice were 2-6 month old in the beginning of the experiments. Mice were housed under a temperature- and humidity-controlled environment (temperature between 21°C and 24°C and humidity between 45% and 50%) with 12h light/12h dark cycle and ad libitum access to water and chow.
Wild animals	No wild animals were used.
Reporting on sex	Both male and female mice were used and reported.
Field-collected samples	No field-collected samples were used.
Ethics oversight	All animal experiments in the current study were performed according to swiss federal guidelines, and had been approved by the Animal Experimentation Committee of the Canton of Zurich under the permits 040/2015, 139/2016, 243/2018 and 236/2019.

Note that full information on the approval of the study protocol must also be provided in the manuscript.
