# Supplementary figures and images for: NG2 glia protect against prion neurotoxicity by inhibiting microglia-to-neuron prostaglandin E2 signaling
Source: Nat Neurosci. 2024 May 27;27(8):1534–44. doi: 10.1038/s41593-024-01663-x (PMC11303249; doi:10.1038/s41593-024-01663-x)

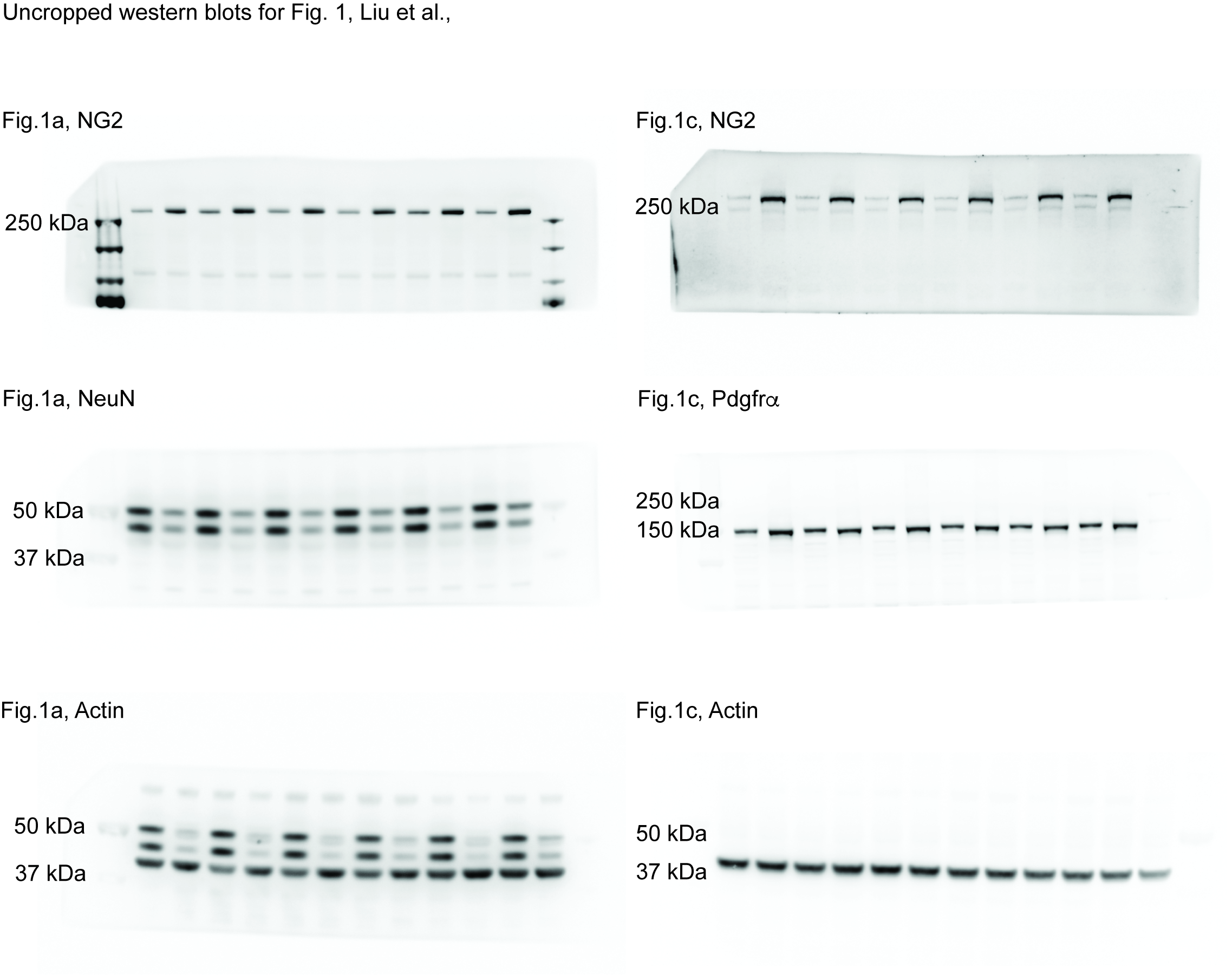

Supplement: Supplementary file 3 — Unprocessed western blots. [file 41593_2024_1663_MOESM3_ESM.tif]

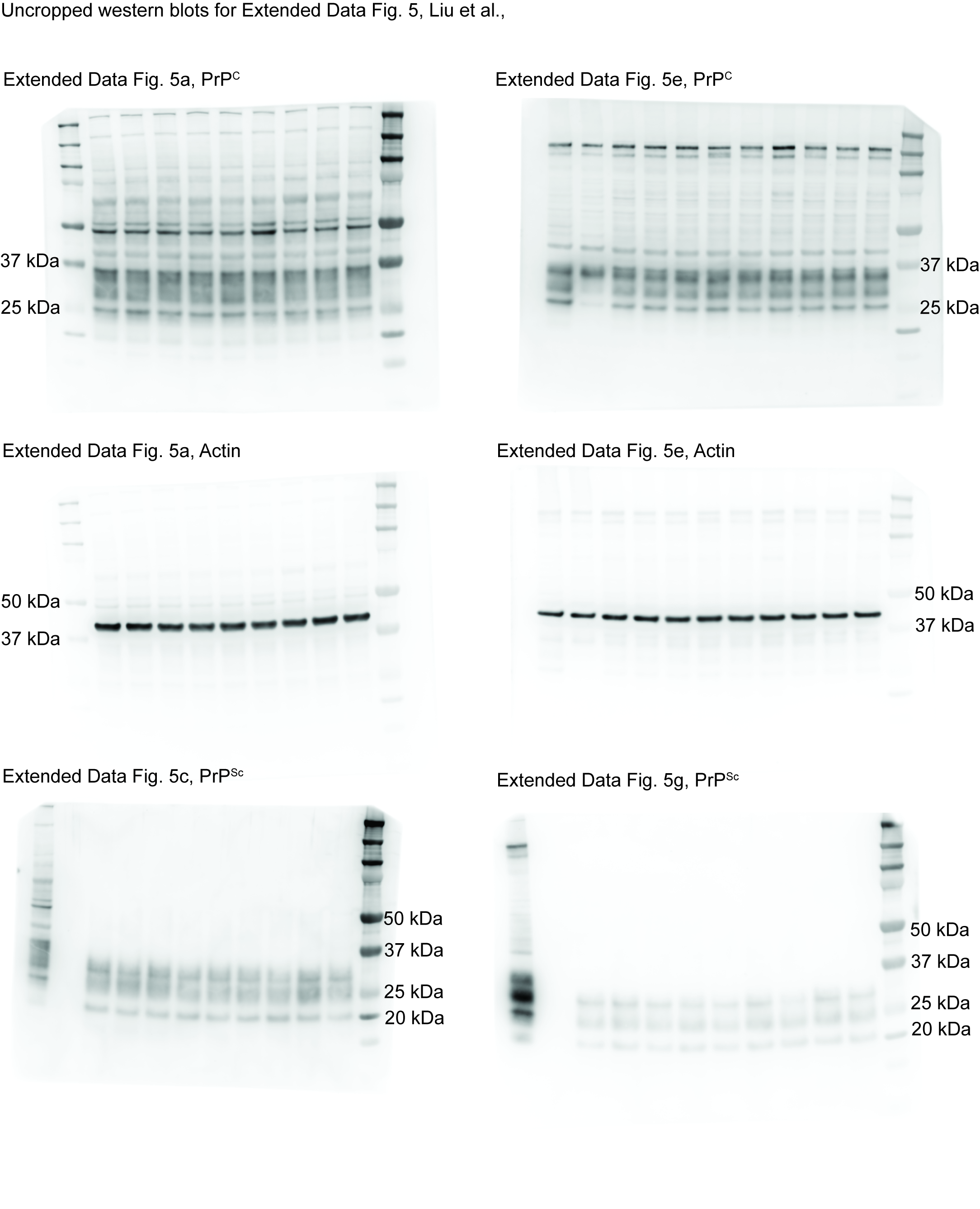

Supplement: Supplementary file 14 — Unprocessed western blots. [file 41593_2024_1663_MOESM14_ESM.tif]
